# Supplementary material for: Polyketide synthase gene clusters in the ericoid mycorrhizal fungus Oidiodendron maius: genome-wide prediction, expression profiling, and functional characterization of knockout mutants
Source: Front Plant Sci. 2026 Sep 16;17:1924430. doi: 10.3389/fpls.2026.1924430 (PMC13623903; doi:10.3389/fpls.2026.1924430)
Supplement: Supplementary file 1 [file SupplementaryFile1.zip › Supplementary Materials/Supplementary Folder 1.zip!/Oidma1_AssemblyScaffolds_Repeatmasked/knownclusterblast/cluster36/jgi.p_Oidma1_131121_mibig_hits.html]

| MIBiG Protein | Description | MIBiG Cluster | MiBiG Product | % ID | % Coverage | BLAST Score | E-value |
| --- | --- | --- | --- | --- | --- | --- | --- |
| AGN71609.1 | amino\_oxidase/esterase | BGC0000027 | Azaphilone\_biosynthetic\_gene\_cluster | 40 | 94.0928270042 | 300 | 2.5e-81 |
| BAK26560.1 | FAD-dependent\_oxidoreductase | BGC0000977 | Cyclopiazonic\_acid\_biosynthetic\_gene\_cluster | 33 | 92.194092827 | 199 | 7.8e-51 |
